# High Humidity Causes Abnormalities in the Process of Appressorial Formation of *Blumeria graminis* f. sp. *hordei*

**DOI:** 10.3390/pathogens9010045

**Published:** 2020-01-05

**Authors:** Koreyuki Sugai, Hiroshi Inoue, Chie Inoue, Mayuko Sato, Mayumi Wakazaki, Kappei Kobayashi, Masamichi Nishiguchi, Kiminori Toyooka, Naoto Yamaoka, Takashi Yaeno

**Affiliations:** 1 Department of Agriculture, Ehime University, Tarumi, Matsuyama, Ehime 790-8566, Japan; 2 RIKEN Center for Sustainable Resource Science, Yokohama, Kanagawa 230-0045, Japan; 3 Research Unit for Citromics, Ehime University, Tarumi, Matsuyama, Ehime 790-8566, Japan

**Keywords:** *Blumeria graminis* f. sp. *hordei*, *Hordeum vulgare*, appressorium, humidity

## Abstract

High humidity decreases the penetration rate of barley powdery mildew *Blumeria graminis* f. sp. *hordei*. However, the mechanism is not well understood. In this study, the morphological and cytochemical analyses revealed that substances containing proteins leaked from the tip of the appressorial germ tube of conidia without the formation of appressorium under a high humidity condition. In addition, exposure to high humidity prior to the formation of appressorium caused the aberrant formation of the appressorial germ tube without appressorium formation, resulting in failure to penetrate the host cell. These findings suggest that the formation and maturation of the appressorium requires a low humidity condition, and will be clues to improve the disease management by humidity control.

## 1. Introduction

Powdery mildew is a major fungal disease of barley, caused by *Blumeria graminis* f. sp. *hordei* (*Bgh*). An obligate biotrophic pathogen *Bgh* can make a success of the asexual life cycle only on the living host cells, resulting in the spread of airborne conidia through the formation of conidiophores. After the arrival to the leaf epidermis, the primary germ tube (PGT) emerges within 2 h. Although PGT does not penetrate the host cell wall, it is hypothesized that PGT has roles not only in the transduction of host-derived signals to initiate the coming morphogenesis and the suppression of host resistance, but also in the absorption of water from host cells under dry condition [1,2,3]. A secondary germ tube starts to develop 4–6 h post inoculation and differentiates into an appressorial germ tube (AGT) 8–10 h post inoculation. The tip part of the AGT undergoes maturation and formation of a hook-structured appressorium about 2 h after the formation of the AGT. To penetrate the cell, the appressorium mounts the formation of a penetration peg at the side contacting with the host surface, using the mechanical force by turgor pressure and the enzymatic degradation of the cell wall [4]. In case of failure to penetrate it through the appressorium, *Bgh* forms the second lobe from the lateral part of AGT as another attempt to penetrate a neighbor cell. Successfully penetrating *Bgh* forms a haustorium surrounded by a host-derived extrahaustorial membrane and establishes infection. Subsequently, the fungus develops secondary hyphae by absorbing nutrients with the haustorium and forms conidiophores to produce new conidia. Because the entire life cycle is accomplished on the surface of host leaves, it is assumed that the infection processes are largely affected by conditions surrounding the leaves (e.g., light intensity, photoperiod and humidity condition) [5,6]. Of particular note is that the effects of moisture differ depending on the infection stages of *Bgh*. For example, as with other fungi, high humidity is required for the sporulation and the germination of conidia [7,8]. However, water-soaked condition, which seems to mimic exposure to rain water, makes *Bgh* more likely to fail the normal development of the germ tubes [9,10,11]. Because the mechanism by which humidity conditions influence each infection process remains to be elucidated, it is necessary to perform in-depth morphological and physiological studies on the relationship between humidity and the development of the germ tube. In this study, we found that the exposure to high humidity prior to the formation of appressorium caused the aberrant formation of the AGT and the leakage of proteinaceous substances from the tip of the AGT. These findings suggest that an appropriate moisture condition is required for the formation of the appressorium of *Bgh*.

## 2. Results and Discussion

### 2.1. Reduction of the Penetration Rate of B. graminis f. sp. hordei under the High Humidity Condition

**Figure 1:**
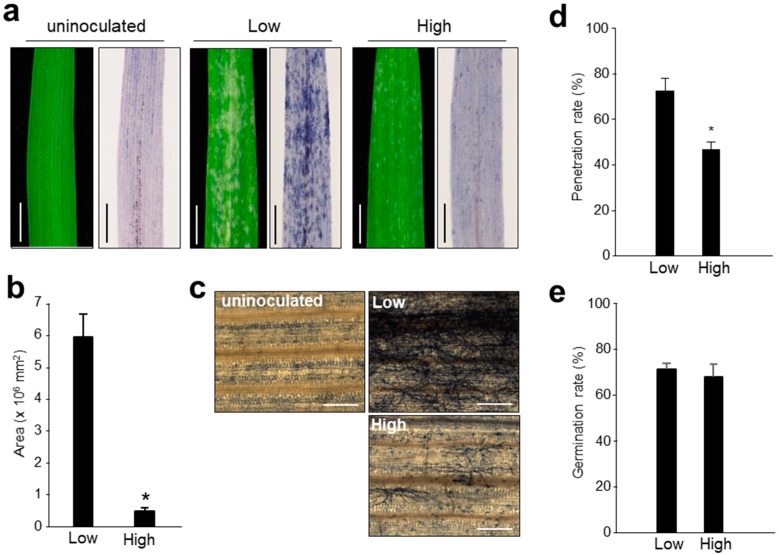
Penetration rate of *B. graminis* f. sp. *hordei* under high humidity condition. (**a**) The barley leaves inoculated with *Bgh* were incubated at about 70% relative humidity (RH, low) or more than 95% RH (high) for 4 d, and then the leaves were stained with trypan blue solution. The uninoculated leaf incubated under low humidity condition was shown as a control. The bars indicate 5 mm. (**b**) Using ImageJ software, the stained mycelia were measured as an area. Data represent means with standard deviation (*n* = 3). The asterisk indicates a significant difference between these conditions (*p* < 0.01; Student’s t test). (**c**) The enlarged photographs of the stained mycelia were taken under a microscope. The uninoculated leaf incubated under low humidity condition was shown as a control. The bars indicate 0.5 mm. (**d**) After inoculation with *Bgh* conidia, coleoptiles were kept at the low or high humidity condition for 24 h. The penetration rate was calculated by counting the number of conidia developing a haustorium per the number of interactions between a barley epidermal cell and a conidium attacking the cell with an appressorium. For each experiment, 15–30 interactions were counted. Data represent means with standard deviation (*n* = 5). The asterisk indicates a significant difference between these conditions (*p* < 0.05; Student’s t test). (**e**) The germination rate was calculated on coleoptiles under both humid conditions. For each experiment, 100–300 conidia were counted. Data represent means with standard deviation (*n* = 6). These experiments were repeated three times with similar results.

When barley leaves inoculated with *Bgh* were exposed to the high humidity condition (more than 95% relative humidity (RH)), the infection was considerably suppressed (Figure 1a–c). Microscopic observation found that some *Bgh* could sporulate, but many could not even form secondary hyphae. Therefore, to examine in detail whether the penetration process is affected by the humid condition, the coleoptiles were kept at about 70% RH (low humidity) or more than 95% RH (high humidity) after inoculation with *Bgh* conidia. The penetration rates were measured 24 h after inoculation. As a result, the penetration rate was significantly reduced under the high humidity condition (Figure 1d). On the other hand, there was no difference in the germination rate between these conditions (Figure 1e). These results suggest that some processes after germination were involved in the suppression of infection under high humidity condition.

### 2.2. Proteinaceous Leakage from the Tip of Appressorial Germ Tube under the High Humidity Condition

**Figure 2:**
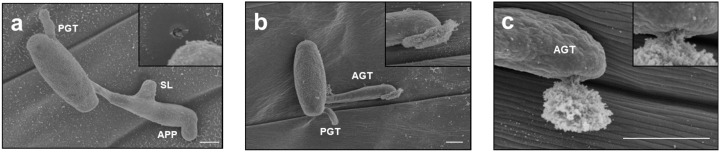
Substances leaked from the tip of the appressorial germ tube (AGT) under the high humidity condition. (**a**) The conidium successfully penetrated a barley epidermal cell under the low humidity condition. The enlarged picture right above shows a penetration pore underneath the second lobe. (**b**,**c**) The conidia had unidentified substances at the tip of AGT under the high humidity condition. APP; appressorium, PGT; primary germ tube, SL; second lobe. Bar = 5 μm.

**Figure 3:**
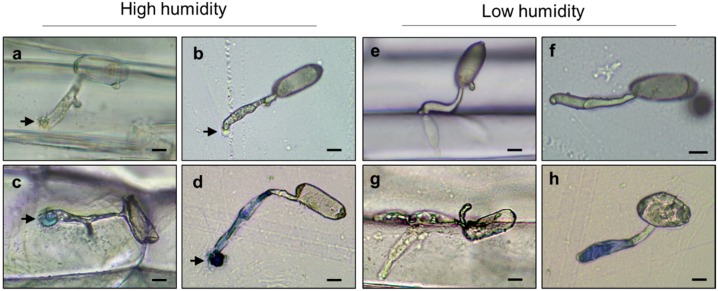
Proteinaceous leakage from the tip of the appressorial germ tube (AGT) under the high humidity condition. Conidia were inoculated onto coleoptiles (**a**,**c**,**e**,**g**) or cellulose membranes (**b**,**d**,**f**,**h**) and incubated under the high or low humidity condition. After incubation, inoculated conidia were stained with Coomassie Brilliant Blue solution for 5 min (**c**,**d**,**g**,**h**). Arrows indicate the substances leaked from the tip of AGT. Bar = 10 μm. These experiments were repeated three times with similar results.

In order to investigate the influence on morphogenesis of *Bgh* by the high humidity condition, a scanning electron microscope analysis was performed. *Bgh* successfully penetrated the cell wall of the barley epidermal cell under the low humidity condition. Figure 2a showed that the second lobe successfully made a penetration pore in the host cell wall although the first attempt to penetrate it from the appressorium was unsuccessful. On the other hand, conidia having unidentified substances at the tip of AGT were noticeable under high humidity condition (Figure 2b). Some were observed as if the substances spouted from the tip of AGT (Figure 2c). The substances were observed in four out of 40 conidia under the low humidity condition and in 13 out of 38 under the high humidity condition. The data, although not statistical, suggested that some kind of substance might be easy to leak from the tip of AGT under the high humidity condition. The appressorium of *Bgh* is supposed to secrete various proteins such as cell wall degrading enzymes, effector proteins for suppressing the penetration resistance of plants and proteins for forming a haustorium. To examine whether the leaked substances contain proteins, Coomassie Brilliant Blue (CBB) staining was carried out after incubation under the high humidity condition. The barley coleoptiles were inoculated with *Bgh* conidia and kept at more than 95% RH for 4 h. The inoculated coleoptiles were transferred to the incubator kept at about 70% RH and then stained with CBB solution 20 h later. As a result, the leaked substances were observed at the tip of AGT and stained blue (Figure 3a,c). To eliminate the possibility that the blue-stained substances were derived from barley epidermal cells, the germinated *Bgh* on the cellulose membranes under the high humidity condition were stained with CBB solution. Likewise, the leaked substances were observed and stained blue (Figure 3b,d). On the other hand, conidia with the leaked substances were not much observed both on coleoptiles and cellulose membranes under continuous 70% RH condition (Figure 3e–h). These results suggest that the stained substances leaked from the tip of AGT and contained proteins and that the high humidity condition during AGT formation caused the proteinaceous leakage.

### 2.3. High Humidity Influences the Formation of the Appressorium

**Figure 4:**
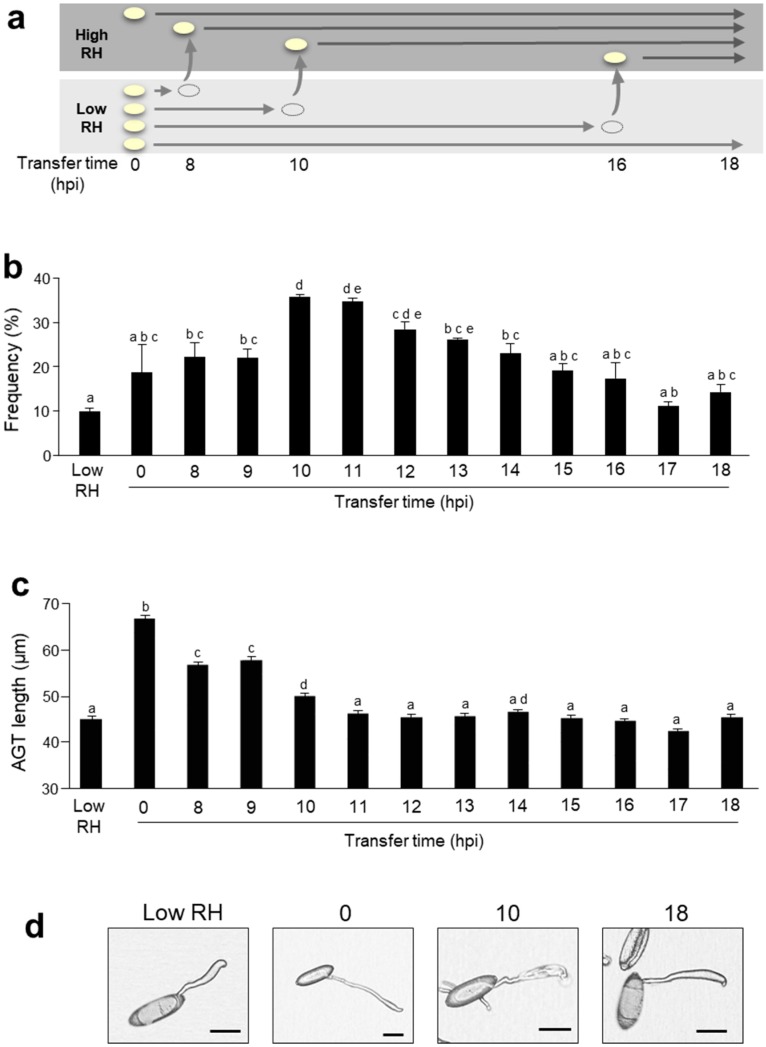
High humidity influences the formation of the appressorium. (**a**) The experimental scheme transferring conidia from low to high humidity condition. (**b**) Frequency of conidia with the leakage at the tip of the appressorial germ tube after transfer to the high humidity condition. After transfer, 200–300 conidia were counted at each time point and the frequencies of the leakage were calculated. Data represent means with standard deviation (*n* = 3). Different letters indicate significant differences (*p* < 0.05, Tukey–Kramer test). (**c**) Length of AGT after transfer to high humidity condition. Data represent means with standard deviation (*n* = 600–1000). Different letters indicate significant differences (*p* < 0.05, Tukey–Kramer test). (**d**) The representative conidia at the timing of each transfer were shown. hpi; hour post inoculation. Bar = 20 μm. These experiments were repeated three times with similar results.

To investigate which timing of the formation process of AGT is involved in the leakage, we analyzed the occurrence frequency of the leakage when conidia were transferred from the low humidity condition to the high humidity condition at various timings of *Bgh* morphogenesis. Conidia were inoculated onto cellulose membranes and the number of conidia with the leakage at the tip of AGT were counted using the optical microscope because the host defense responses, including the cell wall composition, might make the effects of humidity on the *Bgh* morphogenesis variable and it was difficult to obtain as much data as possible statistically analyzed by SEM. The inoculated conidia were transferred to more than 95% RH several hours after incubation at 70% RH (Figure 4a). As a result, the occurrence frequency of conidia with the leakage rose drastically after 10 h of the transfer of conidia and decreased gradually (Figure 4b). Since *Bgh* begins to form the appressorium around 10 h after inoculation, the leakage of proteinaceous substances from the tip of AGT may occur due to the influence of high humidity at the time of the appressorium formation. In fact, the leakage was frequently observed in the AGT that did not yet form a mature appressorium (Figure 2). After the appressorium has been formed, the leakage may be difficult to occur even if conidia are exposed to the high humidity condition. Although no noticeable increase in the occurrence frequency was observed before 10 h of transfer of conidia, the AGT tended to grow longer and spindly when the timing of the transfer was earlier (Figure 4c,d). When transferred to the high humidity condition after 10 h of inoculation, the length of AGT was comparable to that under the low humidity condition. Our results were consistent with previous reports that the abnormal elongation of AGT also occurs when conidia are immersed in liquid [9] and that the AGT is more elongated on the cellulose membrane having high moisture permeability [12]. When exposed to the high humidity condition before 10 h of inoculation, the AGT does not develop normally, hence proteins for the formation of appressorium and the penetration cannot be accumulated, and the leakage of proteinaceous substances may no longer occur. Taken together, high humidity exerts a negative influence on the development of AGT and causes the leakage of proteinaceous substances just at the timing of forming an appressorium. Furthermore, based on the previous report that the turgor pressure of AGT increases during the formation [4], it seems plausible that due to the failure of appressorium formation caused by high humidity, the tip of AGT cannot adhere to the host cell, resulting in the leakage.

### 2.4. Conclusions

In this study, it was revealed that high humidity enhances the leakage of substances containing proteins from the tip of AGT during the formation of appressorium, causing the penetration of *Bgh* to fail. Although it is still unknown how high humidity affects *Bgh* at the molecular level, appropriate humidity is certainly required at the timing of forming an appressorium. Because the leakage is supposed to result from the fractured regulatory processes of appressorium formation, transcriptome or proteome analysis might be effective to obtain clues to elucidate which processes are inhibited. In addition, the morphological observations suggest that *Bgh* may secrete some kinds of proteins from the tip of the appressorium when attempting to penetrate host cells. Our findings will contribute not only to the understanding of infection behaviors of *Bgh* in field conditions that are significantly affected by rainy weather but also to the elucidation of the functions of proteins secreted from the appressorium. In fact, the identification of cell wall degrading enzymes, which are thought to be contained in the secretions from the tip of appressorium, has been attempted to be identified previously [4,13]; however, these genes have not yet been found even though the whole genome of *Bgh* had been sequenced [14,15]. Moreover, there is no report on effector proteins secreted from the appressorium, although several candidates for secreted effector proteins (CSEPs) have been reported as effector proteins by omics analyses of the isolated haustoria [16,17,18]. Therefore, the analysis of proteinaceous substances observed in this study may be a clue for the identification of cell wall degrading enzymes or the appressorial effector proteins which are different from haustorial CSEPs.

## 3. Materials and Methods

### 3.1. Host Plant and Fungal Material

Seedlings of barley (*Hordeum vulgare* cv. Kobinkatagi) were grown in vermiculite supplemented with 300-fold diluted HYPONeX (N:P:K = 6:10:5, HYPONeX Japan, Osaka, Japan) in growth chambers (NK system LH-200-RD, Nippon Medical & Chemical Instruments Co., Ltd., Osaka, Japan) at 20 °C under continuous light condition (ca. 23.6 Wm^−2^). Powdery mildew fungus *Bgh* RACE1 isolate was cultured on the barley leaves at 70% relative humidity (RH) in culture chambers (NK system LH-200-RD, Nippon Medical & Chemical Instruments Co., Ltd., Osaka, Japan) as described by Wahara et al. [19]. Coleoptiles were excised from barley seedlings 7 days after sowing, and single-cell epidermal layers of partially dissected coleoptiles were prepared as described previously [20].

### 3.2. Fungal Inoculation

Conidia were collected from barley leaves around 7 days after inoculation and were inoculated onto coleoptiles or cellulose membranes. The coleoptiles were inoculated with fresh conidia with a brush and were floated on 1 mM CaCl_2_ solution for 18 h as described previously [21]. In vitro culture of *Bgh* on cellulose membranes was performed as described [12] with slight modification. The cellulose dialysis membranes were boiled three times to remove sodium azide and were kept in distilled water before use. The membranes were placed onto 0.8% agar supplemented with 1 mM CaCl_2_ and were dried in the clean bench until water drops on the surface of membranes disappeared. After inoculation at a density of 100–200 conidia/mm^2^, the membranes on agar plates were kept at more than 95% RH in the transparent plastic box or 70% RH in the same box without the lid for 18–24 h in the culture chamber to make the high or low humidity condition, respectively. The penetration rate on coleoptiles was calculated by counting the number of conidia developing a haustorium per the number of interactions between a barley epidermal cell and a conidium attacking the cell with an appressorium. The count was performed with an optical microscope (BX53, Olympus, Tokyo, Japan).

### 3.3. Histochemical Staining

*Bgh* on the coleoptiles or the cellulose membranes were soaked in CBB solution (50% ethanol, 10% acetic acid and 0.25% CBB R-250) for 5 min, rinsed with 30% ethanol and gently washed with distilled water three times. Leaf segments inoculated with *Bgh* were stained with trypan blue solution 4 d after inoculation as described by Yaeno et al. [22]. To quantify the mycelial growth as an area, the average index of the stain per unit area was measured using ImageJ software.

### 3.4. Scanning Electron Microscopy Analysis

Coleoptiles inoculated with *Bgh* were fixed and dehydrated with a series of ethanol 75%, 80%, 95%, twice 100%, isoamyl acetate–ethanol (1:1) for 30 min and twice isoamyl acetate for 1 h. Samples were dried with a critical point dryer (EM CPD030, Leica Microsystems, Wetzlar, Germany), coated with platinum, and observed by a scanning electron microscope (SU-1510, Hitachi, Tokyo, Japan) with a secondary electron detector at an accelerating voltage of 5 kV. To observe the penetration pore, *Bgh* conidia with the appressorium or the second lobe were shifted with a micromanipulator (Narishige, Tokyo, Japan) before fixation.

### 3.5. Measurement of the Frequency of Conidia with the Leakage and the Length of AGT

The cellulose dialysis membranes on agar plates were inoculated with conidia and were transferred from 70% RH to more than 95% RH. The germinated conidia were photographed 18 h after inoculation and the conidia with the leakage at the tip of AGT were counted in randomly selected photographs. The length of AGT was measured using software (FlvFs AF_AR630, FLOVEL Co., Ltd., Tokyo, Japan).
